# Probabilistic reasoning about measurements of equilibrium climate sensitivity: combining disparate lines of evidence

**DOI:** 10.1007/s10584-018-2315-y

**Published:** 2018-11-05

**Authors:** Roger M Cooke, Bruce Wielicki

**Affiliations:** 1 Resources for the Future, 1616 P St NW, Washington, DC, USA; 2 NASA Langley Research Center, Science Directorate, 21 Langley Blvd, Hampton, VA 23681 USA

## Abstract

Where policy and science intersect, there are always issues of ambiguous and conflicting lines of evidence. Combining disparate information sources is mathematically complex; common heuristics based on simple statistical models easily lead us astray. Here, we use Bayesian Nets (BNs) to illustrate the complexity in reasoning under uncertainty. Data from joint research at Resources for the Future and NASA Langley are used to populate a BN for predicting equilibrium climate sensitivity (ECS). The information sources consist of measuring the rate of decadal temperature rise (DTR) and measuring the rate of percentage change in cloud radiative forcing (CRF), with both the existing configuration of satellites and with a proposed enhanced measuring system. The goal of all measurements is to reduce uncertainty in equilibrium climate sensitivity. Subtle aspects of probabilistic reasoning with concordant and discordant measurements are illustrated. Relative to the current prior distribution on ECS, we show that after 30 years of observing with the current systems, the 2*σ* uncertainty band for ECS would be shrunk on average to 73% of its current value. With the enhanced systems over the same time, it would be shrunk to 32% of its current value. The actual shrinkage depends on the values actually observed. These results are based on models recommended by the Social Cost of Carbon methodology and assume a Business as Usual emissions path.

## Introduction

Confronted with unwelcome scientific advice, interested parties may seek out, or in some cases even generate, conflicting scientific views to neutralize the unwelcome impact (Oreskes and Conway 2010). Lacking the ability to evaluate the advice, public media striving for balance can unwittingly promote the idea that conflicting advice can simply be ignored. Behind this perspective is a lack of understanding among the general public about the role of disagreement in science. In addition, there is a defective understanding, rooted in the classical statistical methods which most scientific researchers are taught, of how multiple lines of evidence should be combined, as elaborated in Section 2.

The authors’ recent uncertainty decomposition of current and enhanced measurements for equilibrium climate sensitivity (Cooke et al. 2013, 2015, 2016) provides a basis for exploring the effects of conflicting measurements. The future measurement values invoked for this purpose are of course hypothetical but the effects are obtained by conditionalizing a vetted joint distribution for equilibrium climate sensitivity (ECS), the rate of decadal temperature rise (DTR) and rate of change of cloud radiative forcing (CRF) as measured by current and future enhanced observing systems. This analysis profits from the fact that a prior distribution over equilibrium climate sensitivity and theoretical models connecting ECS with DTR and CRF are provided by the US inter-agency memo on the social cost of carbon (IWGSCC 2009, 2013). This enables a fully Bayesian analysis of these complex interlocking measurement platforms which brings many surprising features to light. Relative to the current prior distribution on ECS, we show that after 30 years of observing with the current systems, the 2*σ* uncertainty band for ECS would be shrunk on average to 73% of its current value. With the enhanced systems over the same time, it would be shrunk to 32% of its current value. The actual shrinkage depends on the values actually observed. These results are conditional on the current understanding of the uncertainty of ECS (IPCC 2013; IWGSCC 2009), as well as recent scientific advances in the decomposition of cloud feedbacks, which dominate the uncertainty of ECS, into their individual observable components (Soden et al. 2008; Zhou et al. 2015). To be clear, uncertainty in emissions (including aerosols) and the effect of “slow feedbacks” outside the current SCC paradigm are not taken into account. Whereas this paper focuses on probabilistic interpretations of measurements and their overall impact on ECS uncertainty, another paper (Hanea et al. 2018) uses this model to explore counter-intuitive results more generally. The choice to use climate sensitivity for this example is based on the lack of progress in reducing uncertainty in climate sensitivity in the last 30 years of research (IPCC 2013).

The remainder of this paper is organized as follows: Section 2 reviews combining evidence from the popular point of view and from the simple classical error models. Section 3 describes the current and enhanced measurement platform forming the basis for this analysis. Section 4 illustrates how conflicting measurements can be almost as informative as concordant measurements. Section 5 treats overall uncertainty from different combinations of measurement platforms. A final section gathers conclusions. An appendix provides a mathematical background for the results in Section 4.

## Simple intuitions on combining measurements

Suppose we have one measurement platform for ECS whose sources of error are known and are unbiased. When this platform returns a value for ECS, then the true value for ECS may be either higher or lower according to how the measurement is deflected by its noise. Unable to know the deflection, we intuitively focus on the measured value and ignore the uncertainty. Confronted with the results of two independent measurements, our intuitions are less clear. If the two measurements agree, we tend to see confirmation and feel more confident in the common result. If they strongly disagree, the effect is often to temporize and await more evidence. Such slow deliberative thinking (Kahneman 2011) is often praised as cautious, in contrast to precipitously acting on impulse. However, in cases where decisions cannot be postponed, we need probabilistic thinking. In the simple statistical error model which most practitioners have learned, the measurements would be modeled as perturbed by independent identically distributed additive error terms. The estimate minimizing mean square error is the mean of the observations and the variance of the estimate is the variance of a single error term divided by the number of observations,^1^ regardless whether the measurements are concordant or discordant.

The intuition that concordant measurements should confer more confidence than discordant measurements is not attested by the simple error model most practitioners know. There are many other examples illustrated in Section 4. This simple error model cannot account for “negative learning” where we become more uncertain after retrieving a measured value than we were before (Oppenheimer and O’Neill 2008; Hanea et al. 2018). Two measurements may return the same values but with different noise, resulting in different predictions. Two measurements may separately produce the same prediction, yet result in a different prediction when combined. Two strongly conflicting measurements may jointly yield a great deal of information about the unknown quantity.

It is common to attribute such divergence between intuitions and simple error models to a difference between classical and Bayesian approaches. Indeed, the features mentioned in the previous paragraph can be ascribed to the interaction between measurement error and a prior distribution on the variable of interest. Bayesian nets are used to illustrate the complexities of combining measurements. However, the appendix shows that the distinction between classical and Bayesian methods is more apparent than real in the contexts of multiple measurement platforms with well-defined error properties: The key idea is that an unknown variable of interest *X* can be modeled as *Z + e* where *Z* is the unknown measured value and *e* is the error with a known distribution. Upon measuring *Z = z*, *X* can be ascribed the distribution of *z + e*. Subsequent measurements can be seen as updating this “prior.” This ascription cannot be described as probabilistic conditionalization as *Z* does not have a distribution, but it can be described as “Renyi conditionalization” (Renyi 1970). Alternatively, we can simply compute the conditional error distributions given the observed values and arrive at the same results without ascribing a distribution to *X*. The two approaches are equivalent. The appendix gives details and provides a simple mathematical model which mimics the results in Section 4 on concordant and discordant measurements.

Neither the simple error model nor our simple intuitions can do justice to the complexities of probabilistic inference with multiple lines of evidence. Real examples combined with graphical software tools for probabilistic inference can help to hone our intuitions.

## Measuring equilibrium climate sensitivity

An enhanced Earth Observing System (EOS) component *CLARREO* (Climate Absolute Radiance and Refractivity Observatory, Wielicki et al. 2013) uses better calibration than existing systems to observe trends in the decadal rate of global surface temperature rise, and decadal percentage changes in CRF. This is compared with existing systems: For global temperature rise, these are weather satellite infrared spectrometers IASI (Infrared Atmospheric Sounder Interferometer, Hilton et al. 2012), *AIRS* (Atmospheric Infrared Sounder, Aumann et al. 2003), and *CrIS* (Cross Track Infrared Sounder, Strow et al. 2013), abbreviated as IAC. They look at about 1/3 of the Earth’s emitted infrared radiation where CO_2_ and H_2_O absorb radiation in varying levels (low absorption levels see to the surface, high absorption see to 20-km altitude, others see to intermediate depths). They use this to gauge temperature and water vapor vertical profiles from the surface to about 20-km altitude. For reflected shortwave *CRF*, the existing system is the *CERES* system, a broadband radiation budget instrument. It measures total reflected solar energy as a single value, and total emitted thermal infrared energy as a second value (Wielicki et al. 1996). All references to CRF in this paper are to reflect shortwave CRF. Note that cloud radiative forcing or CRF is also often referred to as cloud radiative effect or CRE.

There is no correlation of the uncertainties of the *CERES* and IAC instruments. There is almost no common technology, and their calibration issues are very different. The enhanced EOS *CRF* employs a reflected solar spectrometer with a large 2D detector array (512 by 512 detectors) that uses scans of the sun, moon, and nearby deep space to do calibration and international standards (SI) traceability. It shares no types of components with the IR spectrometer, uses a 2-axis gimbal to point the entire instrument so that the exact same optics path is used for solar, lunar, and earth viewing observations. The IR spectrometer that measures temperature change is an interferometer that uses deep cavity blackbodies (0.9998 emissivity where 1.0 is perfect), three different temperature phase change cells to calibrate temperature of the blackbody to SI standards, a blackbody emissivity monitor, and varies its blackbody temperatures for calibration from 200 to 320 K. The physics of how such instruments would change in orbit has no common element, even the electronics of these instruments are very different.

**Fig. 1:**
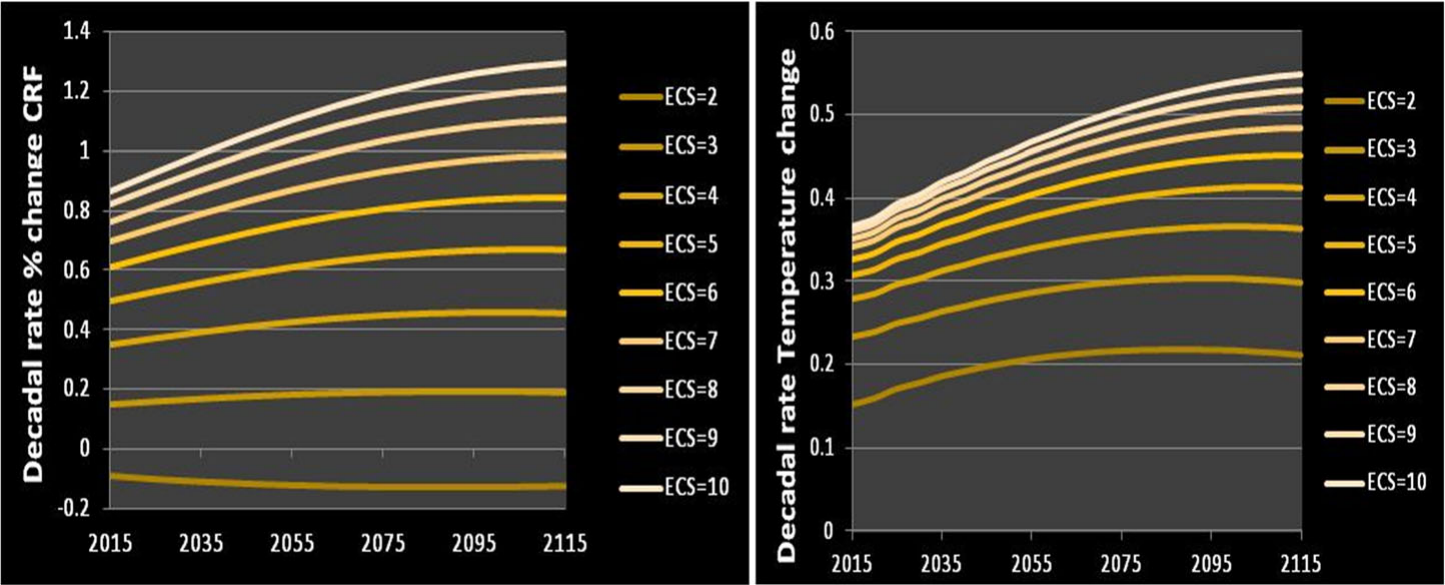
Percentage change in *CRF* (left) and global temperature rise (right) computed with DICE and the Business as Usual emissions path for values of ECS varying ranging from very low (2C) to very high (10C)

Using the integrated assessment model *DICE* (Nordhaus and Sztorc 2013), certified by the Inter-Agency Working Group on the Social Cost of Carbon IWGSCC (2009), theoretical values for decadal temperature and percentage decadal change in *CRF* are determined by *ECS*, the carbon cycle, and the emissions scenario, as shown in Fig. 1. The relationship between ECS and CRF follows the decomposition of climate sensitivity into individual feedback components such as cloud feedback (Soden et al. 2008; Zhou et al. 2015). To be clear, these values are not measured but derived from models. Details on the relationship can be found in Cooke et al. 2016.

The cloud feedback uncertainty in climate models is dominated by low clouds (IPCC 2013). The effect of low clouds on the climate system is in turn dominated by cloud-driven changes in Earth’s reflected solar radiation to space which is typically measured by global mean reflected shortwave CRF (Soden et al. 2008; Zhou et al. 2015). Interannual and decadal changes in shortwave CRF have been shown by climate models to be the key measure of cloud feedback (Soden et al. 2008; Dessler 2010; Zhou et al. 2015, 2016; Zelinka et al. 2017). While we use the simpler framework of Soden et al. (2008) for this demonstration example, newer results have shown that spatial patterns of changes in shortwave CRF can be used to reduce the noise of short-term interannual variability in extracting long-term low cloud feedbacks (Zhou et al. 2016). It has also been shown that use of 500 hPa temperature change in the place of surface temperature change may also reduce the effects of natural variability or short-term climate change (Dessler et al. 2018; Dessler and Forster 2018). In the future, both the spatial pattern effects and 500-hPa temperature changes could be incorporated into the framework presented in this paper. Additional climate feedbacks could also be added. We focus on a simpler framework to provide examples of how information from multiple lines of evidence interact.

Global temperature and change in *CRF* are observed against the background of natural variability, whose effects on trend measurements are attenuated by longer observational times. When perturbed by natural variability (*var*), orbit sampling uncertainty (*orbit*) and instrument calibration drift (*cal*) over observation time *t*, the variance *σ*^2^ of the trend estimate is derived from (Leroy et al. 2008):

$$ {\sigma}^2=12{\left(\Delta t\right)}^{\hbox{--} 3}{\left({\sigma^2}_{\mathrm{var}}{\tau}_{\mathrm{var}}+{\sigma^2}_{\mathrm{cal}}{\tau}_{\mathrm{cal}}+{\sigma^2}_{\mathrm{orbit}}{\tau}_{\mathrm{orbit}}\right)}_{.} $$

The units of the one-period variance components *σ*^2^_var,_ *σ*^2^_cal_, and *σ*^2^_orbit_ are the squares of the physical units being measured. The characteristic times *τ*_var_, *τ*_cal_, and *τ*_orbit_ are in years and reflect the serial correlation. Autocorrelation time scales for natural variability are dominated by ENSO (~ 1.5 years), satellite orbit sampling by the averaging time (1 year), and instrument calibration by instrument lifetime, here assumed to be 5 years (Leroy et al. 2008; Wielicki et al. 2013). The units of *σ*^2^ are thus [physical units squared / time squared]. The effects of noise in observing trends are attenuated by longer observation times. The variance components are considered to be independent normal variables with mean zero (for a detailed discussion see Cooke et al. 2013). The statistical formulation is based on an AR(1) process that accounts for short-term climate variability such as ENSO. Longer term climate variability such as Pacific Decadal Oscillation can also be significant and could be included using an AR(2) or other statistical process in future analysis (Brown et al. 2015).

## Discordant and concordant measurements

**Fig. 2:**
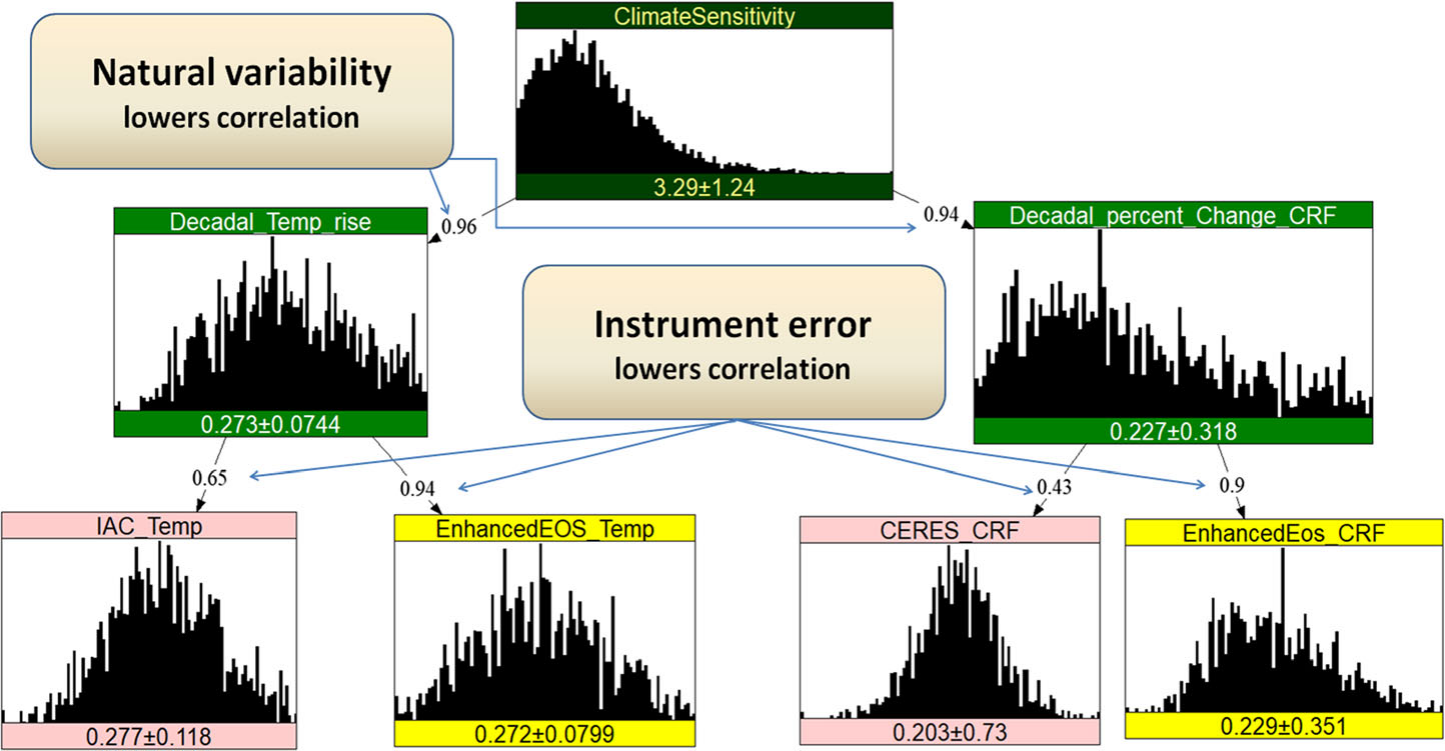
Bayesian Net for combining disparate information; 30 years after launch of Enhanced Earth Observing Systems. “IAC” denotes the weather satellite infrared spectrometers IASI (EUMETSAT instrument), AIRS (NASA instrument), and CrIS (NOAA instrument). The existing *CERES* system is a broadband radiation budget instrument measuring total reflected solar energy and total emitted thermal infrared energy. The “prior” distribution for ECS is the truncated Roe Baker distribution used by the IWGSCC. The unit on the *x*-axes for the histogram for ECS is degrees centigrade [C], for Decadal Temp rise, and for the pink and yellow measurement systems, the *x*-axis is [C] / decade, while for decadal change in CRF, and its pink and yellow measurement systems, the *x*-axis is percentage change in CRF / decade

A Bayesian Net (BN) is a graphical representation of a multivariate probability distribution. The BN software employed here is UNINET,^2^ developed for the Dutch Ministry of Transport. UNINET was designed for non-parametric continuous and discrete variables in very high dimensions (Ale et al. 2009) using (conditional) rank correlations and the normal copula (see Section 5). Rank correlation and the Pearson product moment correlation are typically close, and no distinction is made in this exercise (for details on these and other aspects of UNINET, see Hanea (2008) and Hanea et al. (2015)). If *Z* is an observation of random variable *X* with independent error uncertainty *e* (*X = Z + e*), then the correlation of *Z* and *X* is *σ*_*x*_ / (*σ*^2^_*x*_ *+ σ*^2^_*e*_)^½^, where *σ* denotes the standard deviation. If *X* is a trend, then the error in observing the trend decreases as the trend is observed over a longer time period. *σ*_*e*_ becomes small, the correlation between *Z* and *X* goes to unity and *Z* becomes a perfect measurement of *X* (Leroy et al. 2008). These ratios of standard deviations are used to determine the correlations in Fig. 2 below.

Figure 2 shows the Bayesian net for current (pink) and new (yellow) measurements of the rate of decadal temperature rise (Temp) and percentage rate of change in CRF. Each measurement adds its own instrument uncertainties which, according to the above, are independent. Figure 2 depicts the situation after 30 years before measurements of Temp or CRF. The marginal distributions and correlations are input to the BN which then determines the joint distribution by simulation. Means and standard deviations of the individual variables are shown in the boxes with histograms for each variable. The correlations shown by each arc are determined, as above, by the ratios of standard deviations in the observing and observed systems and the observational time. The joint distribution can then be conditionalized on any set of values of any of the variables. Performing a measurement corresponds to learning a unique value for one or more of the pink and yellow variables. Conditionalization propagates this knowledge through the net thereby reflecting the changes in our uncertainty resulting from the measurement(s).

After 30 years, the trend uncertainty due to natural variability is fairly small and the correlations between ECS and the theoretical trends (green) are high. Greater accuracy with the yellow systems is reflected in higher correlations with the trending variables. The distribution for *ECS* is the truncated Roe Baker distribution used by IWGSCC. The sign convention for CRF decadal change is that positive change indicates increased downward solar energy into the climate system. Units in the figures for temperature trends are given in K/decade and for CRF are given in % CRF/decade.

### Discordant conclusions from concordant measurement results

**Fig. 3:**
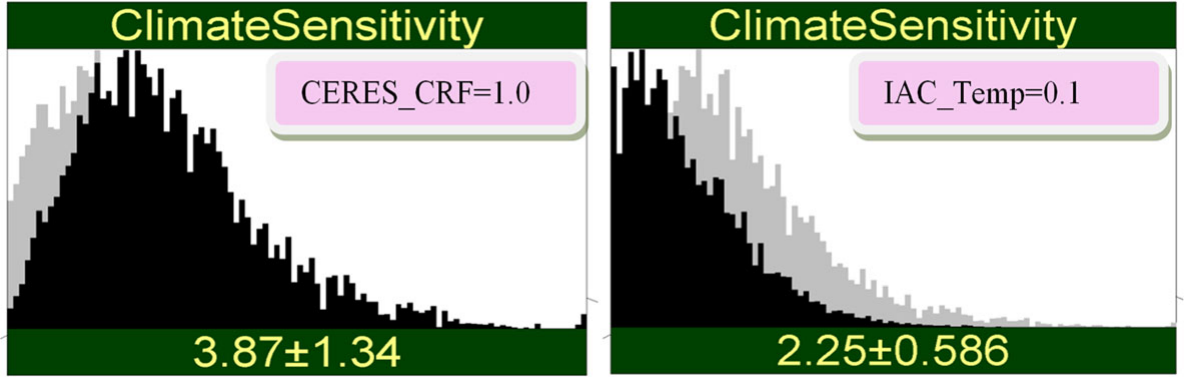
Result of observing a high value (1.0%/decade) with only the CERES_CRF system (left) or observing a low value (0.1 K/decade) with only the IAC system (right). The gray histogram is before measurement; the black histogram is after measurement. The left graphic has higher uncertainty (standard deviation 1.34) than before the measurement (standard deviation 1.24) illustrating negative learning

Probabilistic intuitions are engaged by conditionalizing on possible observed values and propagating this information through the network. For example, suppose we observe a value for CRF (1.0) indicating high ECS with the CERES system. Figure 3 (left panel) shows the result of propagating this information: the new measurement *increases* our uncertainty. The standard deviation of ECS before observing was 1.24, after observing it is 1.34. This “negative learning” (Oppenheimer et al. 2016) is impossible under the simple error model, yet it is very real and can blindside the unwary: The result is greater uncertainty in ECS post measurement than pre-measurement. The results of propagating a value for the IAC Temp (0.1 K/decade) indicating a low value for ECS are also shown in Fig. 3 (right panel). Negative learning does not occur in this case because the prior distribution of ECS is skewed: there is not as much room to maneuver on the low end of ECS values.

**Fig. 4:**
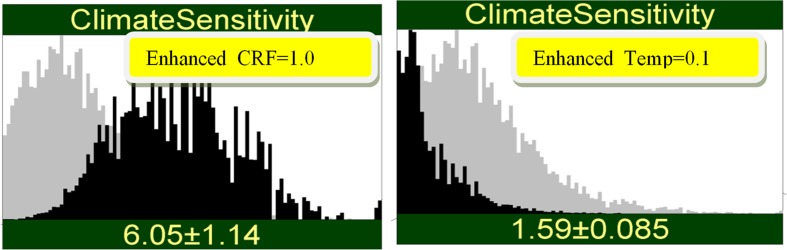
Result of observing a high value (1.0%/decade) with only the enhanced CRF system (left) and observing a low value (0.1 K/decade) with only the enhanced Temp (right). There is no negative learning in this case, because of the lower uncertainty in the enhanced system

If the measured values in Fig. 3 were returned by the enhanced systems, the shift in the distribution of ECS would be much more dramatic (Fig. 4). This is the effect of reducing the error uncertainty in the enhanced measurements. There is no negative learning in this case, yet here again the results would baffle an analyst equipped only with the simple error model. Indeed, how could the pink and yellow measurements return the same numbers yet lead to very different conclusions for ECS? The answer is that with reduced error, the yellow measurements pay less heed to the prior information for ECS. The interactions between measurement error and the prior uncertainty in ECS are complex and easily under-appreciated.

### E pluribus unum

The two measurements in Figs. 4 are strongly conflicting when considered in isolation. Indeed, judging by the resultant expected values for ECS, the enhanced measurements are more discordant than those of the current system. Combining the conflicting results is simply a matter of conditionalizing on both pieces of information. Of course, such conflicting results are unlikely, but if they are observed, we should expect that the enhanced CRF measurement is deflected upward by the noise, while the enhanced_Temp measurement is deflected downward. In other words, given conflicting measured values, the expected errors are negatively correlated (see appendix). This effect is stronger for the enhanced than for the current systems.

**Fig. 5:**
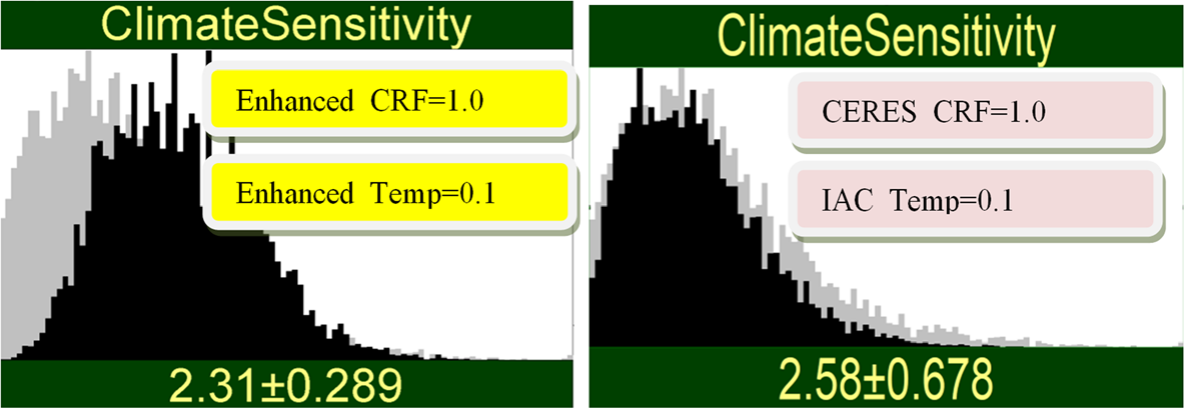
Result of observing both a high value (1.0%/decade) with the enhanced_CRF system and observing a low value (0.1 K/decade) with the enhanced_Temp system (left), and similar information for the IAC and CERES systems (right)

The results of propagating both signals through the network are shown in Fig. 5, for both the current and enhanced systems. In spite of the conflict, synthesizing both signals yields a significant reduction of uncertainty. Note that the current pink systems leave us with greater uncertainty (standard deviation 0.678 versus 0.289) and yields a smaller shift in the mean estimate of ECS (2.58 versus 2.31). Updating the prior of ECS on both pieces of information constrains the posterior distribution of ECS more than one might expect. The more discordant (yellow) measurements induce more than twice the uncertainty reduction in ECS as the more concordant (pink) measurements.

### Ex uno plures

**Fig. 6:**
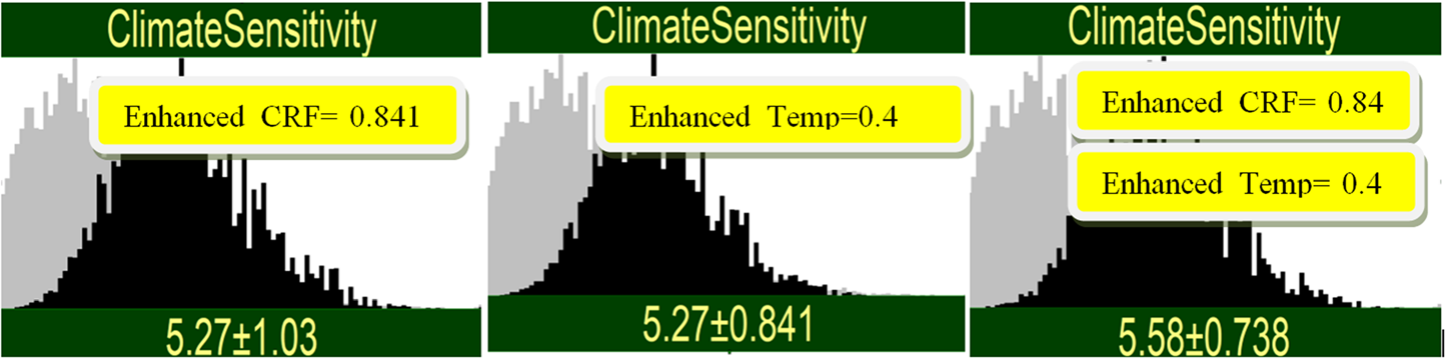
Conditioning on two enhanced measurements with identical estimates of ECS, when combined, yield a higher estimate with lower uncertainty

There is a difference between conflicting and concordant signals, however. If the enhanced_CRF result had been 0.841, and the enhanced Temp result had been 0.4, then each measurement by itself would produce the same mean for ECS, 5.27 (left and middle panels of Fig. 6). However, if the concordant signals are combined, then the re-enforcing effect would raise the mean of ECS from 5.27 to 5.58 and uncertainty in ECS would drop to 0.738. Imagine the scientists saying “your measurement gives the estimate ECS = 5.27, same as mine, so let’s combine our results and estimate ECS = 5.58”. Once again, this is impossible to understand on the simple error model. The explanation lies in the skewed distribution of ECS. The individual measurements would “like” to be higher, but relatively high uncertainties (1.03 for enhanced_CRF, 0.841 for enhanced_Temp) are unable to “drag the prior further upward.” Combining the measurements brings the uncertainty down, allowing the mean value also to rise. The appendix gives a simple mathematical model of this behavior.

Similar behavior will be obtained with lower than expected measured values. If enhanced Temp returns 0.229 and enhanced CRF returns zero, the expectations and standard deviations of ECS, when updated on these values individually, are respectively 0.263 ± 0.406 and 0.263 ± 0.501. If ECS is updated on both measured values simultaneously, the result is 2.56 ± 0.325.

## Overall results

The above results help develop our intuitions for probabilistic reasoning. The ECS estimates themselves depend strongly on the presumed measured values. A more powerful analysis computes the reduction in uncertainty averaged over all possible measurement values. More precisely, we draw a large sample from the joint distribution pictured in Fig. 1. For each value of each measuring platform, and for each vector of values for each combination of measuring platforms, the conditional expectation and conditional standard deviation of ECS are computed. This computation is possible analytically because the BN realizes the correlations using the normal copula. In other words, the marginal distributions pictured in Fig. 1 are assumed to be transformations of standard normal variables. Conditionalization of a joint normal distribution can be performed analytically and the results back-transformed to the original variables. The normal copula imposes features like tail independence and symmetric rank scatter plots. More general copula can be used to avoid these restrictions, at much higher computational expense.

Table 1 shows the results. On average, a measurement of Temp by only IAC reduces the uncertainty (standard deviation) of ECS from 1.24 to 0.96. If we employ only the enhanced system for measuring Temp, we find an average posterior standard deviation of ECS of 0.49. On average, the old (pink) systems provide little uncertainty reduction beyond that gained by the enhanced (yellow) systems. These averages conceal the fact that the old pink systems can still add substantial information in some cases, depending on the actual numbers.

**Table 1:** Posterior average standard deviations of ECS with different combinations of measurement systems, in 2050 following launch in 2020

Average posterior standard deviation ECS, 2050		
Prior to measurement: *μ* = 3.39, *σ* = 1.24C, 2*σ* range = 0.81–5.77[C]		
Temp	IAC	0.96
	Enhanced Temp	0.49
	IAC and enhanced Temp	0.48
CRF	CERES	1.12
	Enhanced CRF	0.63
	CERES and enhanced CRF	0.62
OLD	IAC & CERES	0.90
Enhanced	Enhanced Temp and enhanced CRF	0.41
All		0.40

Table 1 shows that after 30 years of observing with the current systems, the 2*σ* range for ECS would be shrunk on average from its current value of 2.48 to 1.8. In the same time period, the enhanced systems would further shrink the 2*σ* on average range to 0.82. The actual shrinkage will depend on the values of the measurements.

## Conclusion

Enhanced measurements can have economic value only if they are used. By positing a decision context in which society adopts reduced emissions scenarios when high values of equilibrium climate sensitivity are established with requisite confidence, the authors have shown that the real option value of enhanced observation systems, over and above the existing systems, runs into trillions of dollars (Cooke et al. 2015, 2016). This underscores the broader message that probabilistic thinking has economic impact, not just in selecting optimal measurement portfolios, but also in quantifying their social value. While there are many challenges to developing and implementing a more rigorous and accurate long-term climate observing system, the economic benefits suggest that this may be one of the society’s best investments.

Valid probabilistic reasoning is subtle, and trusting untrained intuitions can lead to errors. Scientists, science communicators, policy makers, and general public need to understand that disagreement in science is not dysfunctional but is essential to progress. Cultivating valid probabilistic intuitions through exercises like that performed here hope to promote this understanding.

As final caveat, this study follows the US Interagency Memo on the Social Cost of Carbon. A 2017 study of the National Academies of Sciences (2017) identifies many areas in which the existing methodology can and should be improved. In particular, the state-independent carbon cycle models (as they are called, otherwise known as a system of ordinary differential equations) started at equilibrium cannot reproduce features observed in the data and predicted by Earth system Models of Intermediate Complexity (EMICs).

## Appendix. Conditional expected errors

This appendix contains elementary calculations illustrating features described in Section 4. It also illustrates that in contexts of measurement platforms with well-defined error properties, the distinction between Bayesian and classical statistical approaches is more apparent than real. We consider an unknown quantity *X* observed by variables *Z*_*i*_ = *X* − *e*_*i*_, *i =* 1,2,…*n* where *e*_*i*_ is normally distributed with mean *0* and variance *σ*_*i*_^2^, denoted *N*(0, *σ*_*i*_^2^), and {*e*_1,_ *e*_2_,…*e*_*n*_} are mutually independent. On the classical view, *X* and *Z*_*i*_ do not have distributions. Indeed, the standard definition of the probability that *X = x* given that *Z = z* is *P*(*X = x* AND *Z = z*) */ P*(*Z* = *z*). These probabilities are not defined if *X* and or *Z* do not have a proper distribution. However, if *X* and *Z* are assigned, say, a uniform measure on the real line (which is not normalizable and hence not a distribution), then “Renyi conditionalization” can be defined as the ratios of these two measures.

In any event, the difference *X* − *Z*_*i*_ has the distribution of *e*_*i*_. When (only) *Z*_*i*_ is observed to have value *z*_*i*_, *X* acquires the distribution *N*(*z*_*i*_,*σ*_*i*_^2^). We refer to this operation as “conditionalization on *z*_*i*_”; however, since *Z*_*i*_ does not have a distribution, the designation “Renyi conditionalization” would be more appropriate (Renyi 1970; Cooke 1983). We analyze the information supplied by successive observations as successive conditionalizations of the error distributions. “∝” denotes proportionality and lower case letters *z*_*i*_, *x* denote realizations of *Z*_*i*_ and *X*. We restrict attention to two measurements *Z*_1_, *Z*_2_ but a straightforward generalization yields the case *Z*_1_,…*Z*_*n*_.

If *Z*_*i*_ = *z*_*i*_, *i =* 1,2, then the maximum likelihood estimate of *X* is *x* = z*_1_*w*_1_ *+ z*_2_*w*_2_, where *w*_*i*_ *= σ*_*i*_^−2^/(*σ*_1_^−2^ *+ σ*_2_^−2^). In the classical framework, *X* does not acquire a distribution as a result of these observations, but a distribution is assigned to *x**. Instead, we assign *X* the distribution *N*(*z*_1_, *σ*_1_^2^) upon observing *z*_1_ and proceed to compute the distribution of *X* conditional on *z*_1_, *z*_2_.

Let *f*_*i*_ and *f*_*x*_ denote the densities of *e*_*i*_ and *X*, where *f*_*x*_ is the density of *N*(*z*_1_, *σ*_1_^2^). Compute the mean of *e*_2_ conditional on *z*_1_, *z*_2_ as follows.

A.1 $$ f\left(x,{e}_2\right)={f}_x(x)\times {f}_2\left({e}_2\right). $$

Conditionalize on *z*_2_ by substituting *x = z*_2_ *+ e*_2_:

A.2 $$ {f}_2\left({e}_2\ |\ x,{z}_2\right)\propto \kern0.5em {f}_x\left({z}_2+{e}_2\right)\times \kern0.5em {f}_2\left({e}_2\right)\propto \exp \Big[-\frac{1}{2}\ \left[{e_2}^2/{\sigma_2}^2+{\left({z}_2+{e}_2-{z}_1\right)}^2/{\sigma_1}^2\Big)\ \right] $$

Completing the square, the right-hand side is proportional to

A.3 $$ \exp \Big[-\frac{1}{2}\ \left[{e_2}^2\times \left(1/{\sigma_2}^2+1/{\sigma_1}^2\right)\hbox{--} 2\ {e}_2\left[\ \left({z}_1\hbox{--} {z}_2\right)/{\sigma_1}^2\right]\ \right] $$

which we recognize as a normal density with mean

A.4 $$ E\left({e}_2|\ {z}_1,{z}_2\right)\kern0.5em =\left(\left({z}_1\hbox{--} {z}_2\right)/{\sigma_1}^2\right)/\left(1/{\sigma_1}^2+1/{\sigma_2}^2\right) $$

and variance

A.5 $$ \mathrm{Var}\left({e}_2|\ {z}_1,{z}_2\right)={\left(1/{\sigma_1}^2+1/{\sigma_2}^2\right)}^{-1}. $$

Without imputing the distribution *N*(*z*_1_,*σ*_1_^2^) to *X*, we could perform the same calculation by noting that *e*_1_ *= z*_2_ − *z*_1_ *+ e*_2_ and writing

A.6 $$ f\left({e}_1,{e}_2\right)={f}_1\left({e}_1\right){f}_2\left({e}_2\right)={f}_1\left({z}_2-{z}_1+{e}_2\right){f}_2\left({e}_2\right). $$

Alternatively, we could arrive at the same result by computing *f*_*x*_(*x|z*_1_,*z*_2_) ∝ *f*_*x*_(*x*)*f*_2_(*x* − *z*_2_). The result, intuitively speaking, is that the measurements are “corrected” with the conditional expected errors. The size of the “correction” depends on the distance separating the measured values *z*_1_, *z*_2_, and on the respective unconditional error variances. A direct calculation yields the familiar weighted least squares maximum likelihood estimate as the conditional expectation of *X*.

A.7 $$ E\left(X\ |\ {Z}_1={z}_1,{Z}_2=\kern0.5em {z}_2\right)\kern0.5em =\kern0.5em {z}_1+E\left({e}_1|\ {z}_1,{z}_2\right)={z}_2+E\left({e}_2|\ {z}_1,{z}_2\right)=\kern0.5em \left({z}_1{\sigma_1}^{-2}+{z}_2{\sigma_2}^{-2}\right)/\left({\sigma_1}^{-2}+{\sigma_2}^{-2}\right). $$

The conditional distribution of *X* given *z*_1_, *z*_2_ is the distribution of *z*_1_ *+* (*e*_1_*| z*_1_, *z*_2_) which is the same as *z*_2_ *+* (*e*_2_*| z*_1_, *z*_2_). The difference between the Bayesian and classical approaches reduces to a question of notation.

The expression *E*(*e*_1_*| Z*_1_, *Z*_2_) cannot denote a random variable, as *Z*_1_,*Z*_2_ do not have distributions. However, we can consider it as a function of unknowns *Z*_1_, *Z*_2_. The functions *E*(*e*_1_*| Z*_1_, *Z*_2_), *E*(*e*_2_*| Z*_1_, *Z*_2_) satisfy:

A.8 $$ E\left({e}_1|\ {Z}_1,{Z}_2\right)=\left(\left({Z}_2\hbox{--} {Z}_1\right)/{\sigma_2}^2\right)/\left(1/{\sigma_1}^2+1/{\sigma_2}^2\right)=\kern0.5em -E\left({e}_2|\ {Z}_1,{Z}_2\right){\sigma_1}^2/{\sigma_2}^2. $$

Note that if *Z*_*i*_ *= z*_*i*,_ *i =* 1,2, then *X = z*_2_ *+ e*_2_ *= z*_1_ *+ e*_1_; *e*_2_ *= e*_1_ *+ z*_1_ *- z*_2_, so that conditional on {*Z*_1_ *= z*_1_, *Z*_2_ *= z*_2_}, *e*_1_ and *e*_2_ are perfectly correlated. The situation is as follows. Independently of the distribution of *X*, after observing *z*_1_, *z*_2_, the conditional means of errors *e*_1_, *e*_2_ are no longer *0* but are of opposite sign and proportional to *|z*_1_ *− z*_2_*|*. The conditional distributions of *e*_1_ and *e*_2_ about their respective conditional means each have variance (1/*σ*_1_^2^ *+* 1/*σ*_2_^2^)^−1^ and are perfectly positively correlated.

If *X* has a prior normal distribution with mean *μ* and variance *σ*_*x*_^2^, then a similar calculation yields the conditional expectation:

A.9 $$ E\left(X|{Z}_1={z}_1,{Z}_2={z}_2\right)=\left({z}_1{\sigma_2}^2{\sigma_{\mathrm{x}}}^2+{z}_2{\sigma_1}^2{\sigma_x}^2+{{\mu \sigma}_1}^2{\sigma_2}^2\right)/\left({\sigma_2}^2{\sigma_x}^2+{\sigma_1}^2{\sigma_x}^2+{\sigma_1}^2{\sigma_2}^2\right). $$

The same result would obtain without imputing a distribution to *X* but considering a third measurement *Z*_3_ with *z*_3_ *= μ*, *σ*_3_ *= σ*_*x*_.

If the prior distribution of *X* is not normal, then these simple equations do not hold. Equivalently, suppose the measurements *Z*_*i*_ do not measure *X* directly, but some function of *X*. This is indeed the case in Section 4 where DTR and CRF are functions of ECS. Putting *Z*_*i*_ *+ e*_*i*_ *= ln*(*X*), we can analytically derive features similar to those encountered in Section 4. After observing *z*_1_ the variable *ln*(*X*) is distributed as *N*(*z*_1_, *σ*_1_^2^) and *X* is lognormally distributed. *E*(*X | z*_1_) *=* exp(*z*_1_ + *σ*_1_^2^/2). The conditional expectation of *X* depends on the variance *σ*_1_^2^ and the conditional variance of *X* now depends on the observed value *z*_1_: *V*(*X*) = (exp(*σ*_1_^2^) − 1) × exp(2*z*_1_ + *σ*_1_^2^). If *σ*_1_ *= σ*_2_ and *z*_1_ *= z*_2_, then obviously *E*(*X | z*_1_) *= E*(*X | z*_2_).

To reproduce the behavior found in Section 4, suppose that the prior of *ln*(*ECS*) is distributed as random variable *Z*_*p*_ ~ *N*(0.5, 0.09), *e*_1_ as *N*(0,1) and *e*_2_ as *N*(0,0.943). Let *z*_1_ = 1.5, *z*_2_ = 1.448. Taking the prior distribution of variable *Z*_*p*_ ~ *N*(0.5. 0.09) and adapting (A.9) to the case of a single updating variable, *E*_*Zp*_(ECS | *z*_1_) = 1.7906 and *E*_*Zp*_(ECS | z_2_) = 1.7906. Updating *Z*_*p*_ on both observations gives *E*_*Zp*_(ECS | *z*_1_, *z*_2_) = 1.994.

The counter-intuitive features encountered in the text can easily be explained, within either the Bayesian or classical paradigms, as measuring a nonlinear function of the variable of interest with independent normal error terms.

## Footnotes

1 If the error variances are not equal, then a weighted average is used (see appendix).

2 UNINET is freely downloadable for academic users at http://www.lighttwist.net/wp/uninet. A short video demonstrating the use of UNINET is available at https://youtu.be/NBz5RirkXgw

## References

[CR1] Ale BJM, Bellamy LJ, Boom R, van der Cooper J, Cooke RM, Goossens LHJ, Hale AR, Kurowicka D, Morales O, Roelen ALC, Spouge J (2009). Further development of a Causal model for Air Transport Safety (CATS); Building the mathematical heart. Reliab Eng Syst Saf.

[CR2] Aumann HH (2003). AIRS/AMSU/HSB on the Aqua mission: design, science objectives, data products, and processing systems. IEEE Trans Geosci Remote Sens.

[CR3] Brown PT, Li W, Cordero EC, Mauget SA (2015) Comparing the model-simulated global warming signal to observations using empirical estimates of unforced noise. Nat Sci Rep. 10.1038/srep09957 10.1038/srep09957 PMC4404682 25898351

[CR4] Cooke RM (1983). A result in Renyi’s conditional probability theory with application to subjective probability. J Philos Log.

[CR5] Cooke RM, Wielicki BA, Young DF, Mlynczak MG (2013) Value of information for climate observing systems. Environ, Syst Decis. 10.1007/s10669-013-9451-8

[CR6] Cooke RM, Golub A, Wielicki BA, Young DF, Mlynczak MG, Baize R (2016) Real option value for new measurements of cloud radiative forcing, Resources for the Future, RFFDP 19–16, March 22, 2016

[CR7] Cooke RM, Golub A, Wielicki BA, Young DF, Mlynczak MG, Baize R (2015) Integrated assessment modeling of value of information in earth observing systems. Clim Pol ISSN: 1469–3062 (print) 1752–7457 (online) journal homepage: http://www.tandfonline.com/loi/tcpo20

[CR8] Dessler AE (2010). A determination of the cloud feedback from climate variations over the past decade. Science.

[CR9] Dessler AE, Mauritsen T, Stevens B (2018). The influence of internal variability on Earth’s energy balance framework and implications for estimating climate sensitivity. Atmos Chem Phys.

[CR10] Dessler AE, Forster PM (2018) An estimate of equilibrium climate sensitivity from interannual variability. J Geophys Res Atmos 123. 10.1029/2018JD028481

[CR11] Nordhaus W, Sztorc P (2013) DICE 2013R: Introduction and user’s manual (2nd ed.). http://www.econ.yale.edu/~nordhaus/homepage/documents/DICE_Manual_103113r2.pdf

[CR12] Stocker TF, Qin D, Plattner G-K, Tignor M, Allen SK, Boschung J, Nauels A, Xia Y, Bex V, Midgley PM, IPCC (2013). Climate Change 2013: The Physical Science Basis. Contribution of Working Group I to the Fifth Assessment Report of the Intergovernmental Panel on Climate Change.

[CR13] IWGSCC (Interagency Working Group on Social Cost of Carbon) (2009). Social Cost of Carbon for Regulatory Impact Analysis under Executive Order 12866, Appendix 15a.

[CR14] IWGSCC (Interagency Working Group on Social Cost of Carbon) (2013). Technical support document: technical update of the social cost of carbon for regulatory impact analysis under executive order 12866.

[CR15] Leroy SS, Anderson JG, Ohring G (2008). Climate signal detection times and constraints on climate benchmark accuracy requirements. J Clim.

[CR16] Hanea, A.M., (2008) Algorithms for non-parametric Bayesian Nets Phd Thesis Department Of Mathematics, Delft University Of Technology

[CR17] Hanea AM, Morales Napoles O, Ababei D (2015) Non-parametric Bayesian networks: improving theory and reviewing applications. Reliab Eng Syst Saf. 10.1016/j.ress.2015.07.027 0951–8320/& 2015ElsevierLtd

[CR18] Hanea AM, Nane GF, Wielicki BA, Cooke RM (2018) Bayesian networks for identifying incorrect probabilistic intuitions in a climate trend uncertainty quantification context. Risk Research pp 1–16. 10.1080/13669877.2018.1437059

[CR19] Hilton F (2012). Hyperspectral Earth observations from IASI: five years of accomplishments. Bull Am Meteorol Soc.

[CR20] Kahneman D (2011). Thinking, fast and slow.

[CR21] National Academies of Sciences, Engineering, and Medicine (2017). Valuing Climate Damages: Updating Estimation of the Social Cost of Carbon Dioxide.

[CR22] Oppenheimer M, Little CM, Cooke RM (2016) Expert judgment and uncertainty quantification for climate change, appearing in Nature Climate Change. 6:445–451. 10.1038/NCLIMATE2959

[CR23] Oppenheimer M, O’Neill B (2008). C. and Webster. M. (2008) Negative learning. Clim Chang.

[CR24] Oreskes N, Conway EM (2010) Merchants of doubt: how a handful of scientists obscured the truth on issues from tobacco smoke to global warming, 1st U.S. edn. Bloomsbury Press, New York

[CR25] Renyi A (1970) Theory of probability, North-Holland

[CR26] Soden BJ, Held IM, Colman R, Shell KM, Kiehl JT, Shields CA (2008). Quantifying climate feedbacks using radiative kernels. J Clim.

[CR27] Strow LL (2013). Spectral calibration and validation of the cross-track infrared sounder on the Suomi NPP satellite. J Geophys Res Atmos.

[CR28] Wielicki BA (2013). Achieving climate change absolute accuracy in orbit. Bull Am Meteorol Soc.

[CR29] Wielicki BA, Barkstrom BR, Harrison EF, Lee RB, Smith GL, Cooper JE (1996). Clouds and the Earth’s Radiant Energy System (CERES): an earth observing system experiment. Bull Am Meteorol Soc.

[CR30] Zelinka MD, Randall DA, Webb MJ, Klein SA (2017). Clearing clouds of uncertainty. Nat Clim Chang.

[CR31] Zhou C, Zelinka MD, Dessler AE, Klein SA (2015). The relationship between interannual and long-term cloud feedbacks. Geophys Res Lett.

[CR32] Zhou C, Zelinka MD, Klein SA (2016). Impact of decadal cloud variations on Earth’s energy budget. Nat Geosci.

